# Diverse myeloid cells are recruited to the developing and inflamed mammary gland

**DOI:** 10.1111/imm.13430

**Published:** 2021-11-30

**Authors:** Gillian J. Wilson, Ayumi Fukuoka, Francesca Vidler, Gerard J. Graham

**Affiliations:** ^1^ Chemokine Research Group Institute of Infection, Immunity and Inflammation University of Glasgow Glasgow UK

**Keywords:** chemokine, development, inflammation, mammary, myeloid

## Abstract

The immune system plays fundamental roles in the mammary gland, shaping developmental processes and controlling inflammation during infection and cancer.Here, we reveal unanticipated heterogeneity in the myeloid cell compartment duringdevelopment of virgin, pregnant, lactating and involuting mouse mammary glands,and in milk. We investigate the functional consequences of individual and compoundchemokine receptor deficiency on cell recruitment. Diverse myeloid cell recruitmentwas also shown in models of sterile inflammation and bacterial infection.Strikingly, we have shown that inflammation and infection can alter the abundanceof terminal end buds, a key developmental structure, within the pubertal mammarygland. This previously unknown effect of inflammatory burden during puberty couldhave important implications for understanding pubertal development.

## Graphical abstract

Diverse myeloid cells are recruited to the mammary gland during development, sterile inflammation and bacterial infection. Inflammation and infection can alter developmental structures within the gland.

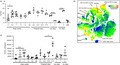

**Abbreviations**
DC: dendritic cell
DM: Ductal macrophage
ECM: extracellular matrix
ECP: *Escherichia coli* particles
iCCR: inflammatory chemokine receptor
LAL: lobuloalveoli
MDSC: myeloid‐derived suppressor cells
NK: natural killer
t_SNE: t‐distributed stochastic neighbour embedding
TAM: tumour‐associated macrophage
TAN: tumour‐associated neutrophils
TEB: terminal end bud

## INTRODUCTION

The mammary gland is a highly regenerative tissue within the body. It is unique, in that most of its development occurs postnatally throughout the female reproductive lifetime. The gland undergoes dramatic structural changes throughout puberty where proliferative structures, terminal end buds (TEB), invade through the surrounding fatty stroma, giving rise to a complex epithelial network in adulthood [1]. During pregnancy, rapid proliferation of epithelial cells generates lobuloalveoli (LAL), and milk producing ducts form during lactation. When lactation stops upon weaning, a process of involution occurs where 90% of the gland remodels to its pre‐pregnancy form [1].

The immune system has long been identified as a key component of the mammary gland, residing within a stromal population containing fibroblasts, extracellular matrix (ECM) and adipocytes [2]. In particular, macrophages play an essential role in regulating mammary gland branching morphogenesis, as development is dramatically impaired in macrophage‐deficient mice [3, 4]. In addition, the density of branching is reduced in CCL11‐deficient mice, which have decreased numbers of eosinophils [4]. Mast cell degranulation is also necessary for normal ductal development [5]. The adaptive immune system plays an inhibitory role in the regulation of pubertal development through CD11c+ antigen‐presenting cells and CD4+ T cells [6]. Neutrophils and dendritic cells (DC) are also key cell types present in the gland during involution [7, 8, 9].

During inflammation, the immune cell landscape of the mammary gland is altered. In breast cancer, immune cells infiltrate the gland, including tumour‐associated macrophages (TAMs), myeloid‐derived suppressor cells (MDSC), tumour‐associated neutrophils (TANs), T cells and NK cells [10]. Mastitis is a common disease of the lactating breast, caused by a build‐up of milk in the ducts and exacerbated by bacterial infection. Increased numbers of leukocytes, including neutrophils, monocytes and macrophages, are detected in the gland and in milk during mastitis [11, 12]. This impacts milk quality, leading to reduced infant weight gain and dysregulated immune development [13] and often leads to early cessation of breastfeeding.

The molecular mechanisms which regulate the movement of immune cells as they infiltrate into the gland to mediate their effects are not fully understood. Insights into these processes will enhance our understanding of how immune cells contribute to mammary gland development and protect against inflammation. Chemokines, characterized by a conserved cysteine motif, are a family of proteins important in cell recruitment and as in vivo regulators of cell movement within tissues. The chemokine family is comprised of CC, CXC, XC and CX3C sub‐families according to cysteine distribution, and chemokines act through G protein‐coupled receptors to facilitate leukocyte migration [14]. Inflammatory chemokine receptors (iCCRs: CCRs1, 2, 3 and 5) are often expressed by immune cells and are required for cell recruitment within the body [15]. Previously we have shown important roles for chemokine receptors in shaping the macrophage dynamics within the mammary gland to control pubertal development [16, 17].

Here, we reveal unanticipated heterogeneity in myeloid cells within the mammary gland at key developmental stages in virgin, pregnant, lactating and involuting mice. We also reveal the myeloid cell composition of murine milk. In addition, we show that diverse myeloid cells are recruited to the mammary gland during local inflammation and remote infection. Importantly, we have shown that inflammation and infection alter the number of TEBs, key developmental structures, within the pubertal mammary gland. This direct effect of inflammatory burden on pubertal development has not been reported previously.

## RESULTS

### Leukocyte levels in the mammary gland throughout development

**FIGURE 1:**
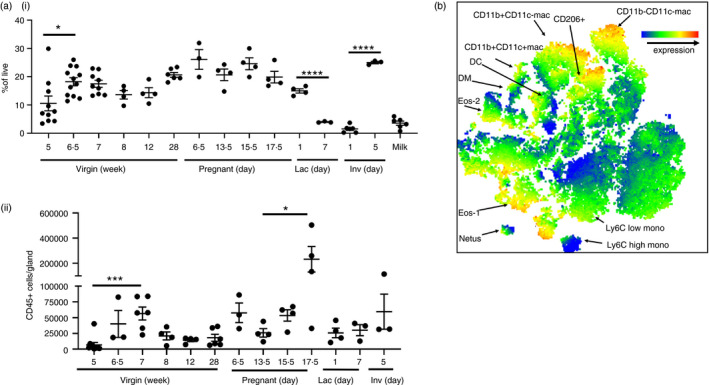
Leukocyte levels in the mammary gland throughout development. a) Flow cytometry of CD45+ cells in the mammary gland expressed as i) a percentage of live cells during virgin development (5 weeks, *n* = 10, 6·5 weeks, *n* = 12, 7 weeks, *n* = 9, 8 and 12 weeks, *n* = 4, 28 weeks *n* = 6), pregnancy (day 6·5, *n* = 3, day 13·5, *n* = 4, day 15·5, *n* = 4, day 17·5, *n* = 4), lactation day 1 (*n* = 4) and 7 (*n* = 3), involution day 1 (*n* = 6) and 5 (*n* = 3), and in milk (*n* = 6). **ii)** Total CD45+ cells per gland, during virgin development (5 weeks, *n* = 10, 6·5 weeks, *n* = 3, 7 weeks, *n* = 6, 8 and 12 weeks, n = 4, 28 weeks n = 6), and pregnancy (day 6·5, *n* = 3, day 13·5, *n* = 4, day 15·5, *n* = 4, day 17·5, *n* = 4), lactation day 1 (n = 4) and 7 (*n* = 3), and involution day 5 (*n* = 3). b) Representative t‐SNE analysis of CD45+ cells within a sample from day 13·5 of pregnancy. The colour scale shows the intensity of F4/80 expression. Statistical analysis was carried out within each developmental stage. Significantly different results are indicated. Error bars represent SEM

To determine the levels of immune cells in the mammary gland at key time points throughout virgin development, pregnancy, lactation and involution, and in maternal milk, flow cytometry was carried out. Leukocytes were defined as CD45‐positive cells and gated as outlined in Supplemental Figure S1. The percentage of CD45+ cells within the live population significantly increased between early (5 weeks) and late (6·5 weeks) puberty (Figure 1ai). CD45+ cells represent a much lower percentage of live cells within the gland on the first day of involution and in milk. The absolute number of CD45+ cells per gland (Figure 1aii) was found to peak during late puberty at 7 weeks in the virgin mammary gland and at day 17·5 during pregnancy. A detailed flow cytometric analysis was carried out to identify myeloid cell types using a defined panel of cell surface markers (Table 1, Supplemental Figures S1 and S2). t‐SNE analysis carried out of CD45+ cells revealed distinct clusters corresponding to the cell types identified (Figure 1b).

**TABLE 1:** Myeloid cells within the mammary gland

Cell Type	Surface marker							
	CD11b	CD11c	F4/80	SiglecF	Ly6C	Ly6G	MHCII	CD206
CD11b+F4/80+ Macrophage	+		+					
CD206+ macrophage			+	−				+
CD11b+CD11c+ macrophage	+	+	+				+	
Ductal macrophage	−	+	+				+	
CD11b−CD11c− macrophage	−	−	+				+	
CD11b+CD11c− macrophage	+	−	+				+	
Ly6C low monocyte	+				Low	−		
Ly6C high monocyte	+				High			
Eosinophil 1	+		+	+				
Eosinophil 2	+		−	+				
Neutrophil	+		−			+		
Dendritic cell		+	−				+	

### Diverse macrophage subsets in the mammary gland throughout development

**FIGURE 2:**
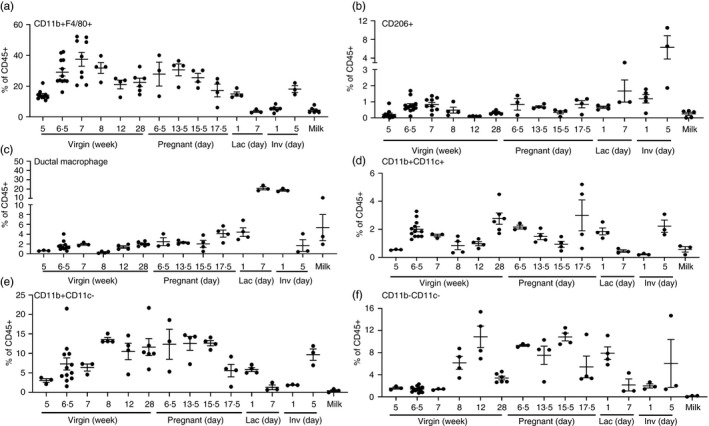
Diverse macrophage subsets in the mammary gland throughout development. Flow cytometry was used to determine the percentage of a) CD11b+F4/80+, b) SiglecF−F4/80+CD206+, c) CD11b−CD11c+MHCII+F4/80+ ductal macrophages, d) CD11b+CD11c+MHCII+F4/80 + e) CD11b+CD11c−MHCII+F4/80+, and f) CD11b−CD11c−MHCII+F4/80+ macrophages within the CD45+ compartment of the mammary gland, during virgin development, pregnancy, involution and in milk. a, b) virgin (5 weeks, *n* = 10, 6·5 weeks, *n* = 12, 7 weeks, *n* = 9, 8 and 12 weeks, *n* = 4, 28 weeks *n* = 6) pregnancy (day 6·5, *n* = 3, day 13·5, *n* = 4, day 15·5, *n* = 4, day 17·5, *n* = 4), lactation day 1 (*n* = 4) and 7 (*n* = 3), involution day 1 (*n* = 6) and 5 (*n* = 3) c–f) virgin (5 weeks, n = 3, 6·5 weeks, *n* = 12, 7 weeks, *n* = 3, 8 and 12 weeks, *n* = 4, 28 weeks *n* = 6), pregnancy (day 6·5, *n* = 3, day 13·5, *n* = 4, day 15·5, *n* = 4, day 17·5, *n* = 4), lactation day 1 (*n* = 4) and 7 (*n* = 3), involution days 1 and 5 (*n* = 3) and milk (*n* = 3). Statistical analysis was carried out within each developmental stage. Significantly different results are indicated in Supplemental Table S1. Error bars represent SEM

In macrophage‐deficient mice, TEB formation in puberty and LAL development in pregnancy are significantly impaired [3, 4]. In this study, CD11b+F4/80+ macrophages increase between early and late puberty, at 5 and 6·5 weeks, respectively (Figure 2a). They also represent a substantial proportion (approx. 20–30%) of the CD45+ cells within the mammary gland through adulthood (8 weeks to 6 months) and in pregnancy. Increased proportions of macrophages are consistent with our understanding of their functions promoting branching at these key developmental stages. CD11b+F4/80+ macrophages represent around 5% of CD45+ cells in the gland during established lactation (day 7), early involution, and in milk. (Figure 2a).

Recent studies have revealed further complexity in the macrophage population within the mammary gland. We identified a small but key population of SiglecF‐F4/80+CD206+ macrophages recruited by CCR1 which promote branching morphogenesis during late puberty [17]. Here, we show a significant increase in this population between early (5 weeks) and late puberty (6·5 weeks) (Figure 2b). They are also detected during pregnancy, lactation and involution but their role in controlling branching at these key timepoints has not been investigated (Figure 2b). In addition, Dawson et al identified and characterized a novel ductal macrophage (DM) important in tissue remodelling during lactation and involution [18]. Notably these macrophages are CD11b negative and further defined as CD11c+MHCII+F4/80+ [18]. Here, we show that DMs represent a low percentage of CD45+ cells throughout virgin development and pregnancy (Figure 2c). However, as described in the original study, they represent a dominant population in lactation and involution [18], comprising around 20% of CD45+ cells in the current study (Figure 2c). Of note, DMs were also found in milk which could imply they have a role in infant immune protection (Figure 2c).

In addition, we have identified three further subsets identified by expression of known macrophage markers F4/80 and MHCII, and differential expression of CD11b and CD11c; CD11b+CD11c+, CD11b+CD11c− and CD11b−CD11c− macrophages (Supplemental Figure S1, Table 1). CD11b+CD11c+ macrophages are a small population which significantly increase during puberty, between 5 and 6·5 weeks, and again during ageing, between 12 and 28 weeks (Figure 2d). They are also detected during pregnancy, lactation and involution, and in milk (Figure 2d). CD11b+CD11c− macrophages are present at each stage of virgin development and pregnancy and at a reduced level during established lactation and in early involution (Figure 2e). CD11b−CD11c− macrophages are found at increased levels in early adulthood and pregnancy (Figure 2f). We also investigated expression of additional macrophage markers CD64 and MerTK by each of the macrophage subsets identified (Supplemental Figure S3). Within each subset, there is a high percentage of CD64+ MerTK− cells and a smaller percentage of CD64+MerTK+cells. The exception to this is CD206+ macrophages which are almost exclusively CD64+MerTK+ (Supplemental Figure S3). Of note, expression of the universal macrophage marker CSF1R is heterogeneous within each subset which may indicate contamination by other cell types. Further studies are required to determine whether these subsets represent different cell types or altered activation states of mammary gland macrophages, and whether they have overlapping or distinct functions within the gland.

### Monocytes, granulocytes and dendritic cells in the mammary gland

**FIGURE 3:**
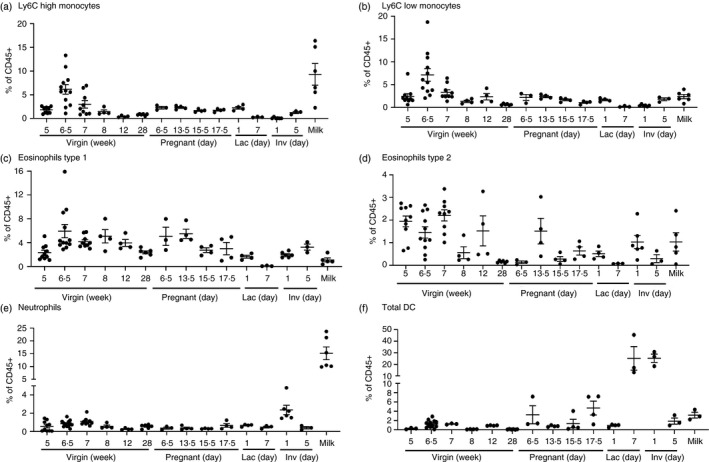
Monocytes, granulocytes and dendritic cells in the mammary gland throughout development. Flow cytometry was used to determine the percentage of a) CD11b+Ly6C high monocytes b) CD11b+Ly6C low Ly6G− monocytes, c) CD11b+SiglecF+F4/80+ type 1 eosinophils d) CD11b+SiglecF+F4/80− type 2 eosinophils e) F4/80−CD11b+Ly6G+ neutrophils and f) F4/80−CD11c+MHCII+dendritic cells, within the CD45+ compartment of the mammary gland, during virgin development, pregnancy, involution and in milk. a–e) virgin (5 weeks, *n* = 10, 6·5 weeks, *n* = 12, 7 weeks, *n* = 9, 8 and 12 weeks, *n* = 4, 28 weeks *n* = 6), pregnancy (day 6·5, *n* = 3, day 13·5, *n* = 4, day 15·5, *n* = 4, day 17·5, *n* = 4), lactation day 1 (*n* = 4) and 7 (*n* = 3), involution day 1 (*n* = 6) and 5 (*n* = 3) and milk (*n* = 6). f) virgin (5 weeks, *n* = 3, 6·5 weeks, *n* = 12, 7 weeks, *n* = 3, 8 and 12 weeks, *n* = 4, 28 weeks *n* = 6) pregnancy (day 6·5, *n* = 3, day 13·5, *n* = 4, day 15·5, *n* = 4, day 17·5, *n* = 4), lactation day 1 (*n* = 4) and 7 (*n* = 3), involution days 1 and 5 (*n* = 3) and milk (*n* = 3). Statistical analysis was carried out within each developmental stage. Significantly different results are indicated in Supplemental Table S1. Error bars represent SEM

We next examined the presence of further myeloid cell types in the mammary gland using the gating strategy outlined in Supplemental Figure S2 and Table 1. There are 2 populations of monocytes in the mammary gland, Ly6C high and Ly6C low, which increase in late puberty (Figure 3a,b). Previously we have shown that monocytes recruited by CCR2 are not required for ductal outgrowth during puberty; however, they were involved in regulating TEB and branch morphology [16]. Monocytes are also found in adult virgin, lactating and pregnant glands, and at low levels during early involution but their role in controlling branch morphology at these stages is not known (Figure 3a,b). In milk, both subsets were identified, with particularly high levels of Ly6C high monocytes, which could suggest a role for these key innate immune cells in infant immune protection. Eosinophils are known to play an important role in regulating the density of branching during puberty [4]. Here, we reveal there are 2 distinct populations of eosinophils characterized by F4/80 expression. Type 1 eosinophils (F4/80+) represent a higher percentage of CD45+ cells than type 2 (F4/80−) (Figure 3c,d). Within the type 1 eosinophil population, there appears to be further heterogeneity with high and low expression of F4/80 (Supplemental Figure S2c). Both are detected in adult, pregnant and involuting glands and in milk (Figure 3c,d). The percentage of type 1 but not type 2 eosinophils increases during late puberty (Figure 3c,d). Notably, reduced levels of monocytes and eosinophils are detected within the mammary gland as lactation progresses (Figure 3). As has been reported previously, neutrophils are found in the mammary gland during involution [9, 19] and represent a substantial proportion, around 15%, of leukocytes in murine milk (Figure 3e). To our knowledge, this is the first description of neutrophils present at low levels throughout virgin and pregnant gland development, and their role at each of these stages is unknown (Figure 3e). Similarly, we have identified small numbers of dendritic cells (DCs) throughout virgin and pregnant development, which dramatically rise during lactation and involution (Figure 3f). DCs have previously been described in lactating and involuting glands [7]. DCs are also detected in milk which could suggest they are important for infant immunity (Figure 3f). To investigate heterogeneity within the total DC population, we included the surface markers CD11b and CD103 (Supplemental Figure S4). There are 4 subpopulations CD11b−CD103+, CD11b+CD103+, CD11b−CD103− and CD11b+CD103− present in the mammary gland throughout development, as have been described previously [7]. Notably CD11b−CD103− DCs predominate in the mammary gland during lactation (Supplemental Figure S4) [7].

CSF1R is a marker for cells of the mononuclear phagocyte lineage including macrophages, monocytes and granulocytes (Sasamono et al, 2003). We carried out flow cytometry of *MacGreen* (CSF1R GFP reporter) transgenic mice to confirm that each of the cell types we have identified in the mammary gland is of myeloid origin (Supplemental Figure S5). In addition, as some immune cell populations are known to change with oestrous stage [20], we compared the cell types studied here between proestrus, oestrous and metestrus/diestrous and found no significant changes (Supplemental Figure S6).

### Inflammatory chemokine receptors are required for cell recruitment to the mammary gland

**FIGURE 4:**
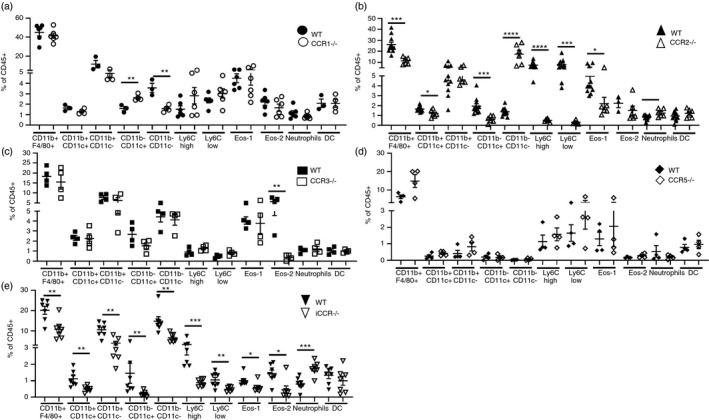
iCCRs promote cell recruitment to the mammary gland. Flow cytometry was used to determine the percentage of CD11b+F4/80+ macrophages, CD11b+CD11c+, CD11b+CD11c−, CD11b−CD11c− and ductal macrophages, Ly6C high and Ly6C low monocytes, type 1 (eos‐1) and 2 (eos‐2) eosinophils, neutrophils and total DCs within the CD45+ compartment of the mammary gland during virgin development of a) WT (black circles) and CCR1−/− (white circles) (7 weeks, *n* = 6 per group, except DCs, CD11b+CD11c+, CD11b+CD11c−, CD11b−CD11c− and ductal macrophages WT *n* = 3, CCR1−/− *n* = 4), b) WT (black triangles, 7 weeks, *n* = 11) and CCR2−/− (white triangles, 7 weeks, *n* = 6), c) WT (white squares) and CCR3−/− (black squares) (7 weeks, *n* = 4 per group), d) WT (white diamonds) and CCR5−/− (black diamonds) (12 weeks, *n* = 4 per group), c) WT (white triangles) and iCCR−/− (black triangles), (7 weeks, *n* = 7 per group). Significantly different results are indicated. Error bars represent SEM

To investigate which iCCRs are required for the recruitment of myeloid cells to the mammary gland, we analysed key cellular populations in WT, individual receptor‐deficient mice and mice with a compound receptor deletion of all four iCCRs, CCRs1, 2, 3 and 5. The receptor‐deficient mouse strains are on different genetic backgrounds and therefore have been compared with their appropriate WT. Previously we revealed that CD206+ macrophages are reduced in CCR1‐deficient mice during late puberty (7 weeks) which leads to delayed branching in the gland [17]. Here, we reveal that CCR1 deficiency also leads to an increase in ductal macrophages and a decrease in CD11b−CD11c− macrophages (Figure 4a). In the absence of the key monocyte receptor CCR2, both Ly6C high and Ly6C low monocytes are depleted in 7‐week‐old mice (Figure 4b). We also observed a reduction in type 1 eosinophils, CD11b+CD11c+ and ductal macrophages (Figure 4b). Previously we observed that CD11b+F4/80+ cells were unaffected in adult CCR2−/− mice [16]; however, in this study we observe a reduction in CD11b+F4/80+ cells during puberty (Figure 4b). As we and others have reported previously, CCR2 deficiency does not impair ductal branching, although there are minor alterations to TEB and branch morphology [16, 21]. In CCR3−/− mammary glands, there is a significant reduction in type 2 eosinophils but not in any of the other populations investigated. (Figure 4c). In the absence of CCR5, we observed no differences in cell recruitment (Figure 4d). Importantly, in pubertal iCCR−/− mice which lack all 4 receptors, we also observed significant reductions in Ly6C high and Ly6C low monocytes, type 1 and 2 eosinophils, CD11b+F4/80+, CD11b+CD11c+, CD11b+CD11c−, ductal macrophages and CD11b−CD11c− macrophages (Figure 4e). This recapitulates the results of the individual receptor‐deficient mice and suggests there are no additional combinatorial effects of compound receptor deficiency on cell recruitment (Figure 4d) or, as we have previously shown, on ductal branching [17].

### Myeloid cells are recruited to the mammary gland during inflammation and infection

**FIGURE 5:**
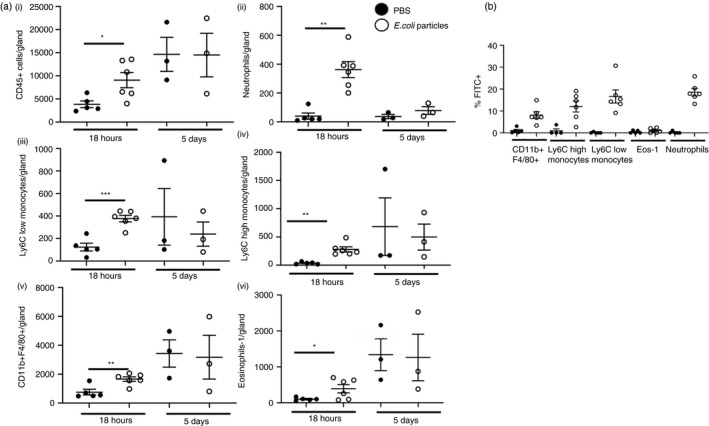
Myeloid cells are recruited to the mammary gland during sterile inflammation. Flow cytometry was used to determine a) the number of i) CD45+ cells, ii) neutrophils, iii) CD11b+F4/80+ macrophages, iv) Ly6C low monocytes, v) Ly6C high monocytes and vi) type 1 eosinophils (eos‐1) within the mammary gland after subcutaneous challenge with either PBS (denoted by black circles) or 500 µg of FITC labelled *E*. *coli* particles (ECP) (white circles), for 18 h (PBS, *n* = 5, ECP, *n* = 6) and 5 days (*n* = 3 per group). b) The percentage of cells bound by FITC labelled ECP 18 h after challenge (PBS, *n* = 5, *E*. *coli* particles, *n* = 6). Significantly different results are indicated. Error bars represent SEM

To investigate the immune response in the virgin mammary gland during local inflammation, we injected mice during late puberty (6–7 weeks), subcutaneously at the site of the mammary gland with either PBS or 500 µg of FITC labelled *Escherichia coli* particles (ECP) for 18 h and 5 days. Cell recruitment to the underlying mammary gland was measured by flow cytometry. Overall, the number of CD45+ leukocytes significantly increased 18 h after challenge with ECP, with no difference observed in the resolution phase, after 5d (Figure 5a). Specifically, neutrophils, Ly6C high and Ly6C low monocytes, CD11b+F4/80+ macrophages and type 1 eosinophils are all increased in the mammary gland 18 h after challenge with ECP (Figure 5a). In addition, we observed binding of the FITC labelled ECP to each of these cell types after 18 h (Figure 5b). After 5 days, the number of cells was not significantly different and bound FITC labelled ECP were not detected (Figure 5). We observed no change in recruitment of, or ECP binding, to ductal CD11b+CD11c+, CD11b+CD11c−, CD11b−CD11c− or CD206+ macrophages, type 2 eosinophils or dendritic cells. Of note, there is an increase in CD45+ cells in PBS challenged mice between 18 h and 5 days after injection. This likely reflects natural increases in immune cells during puberty as has been described in this study and our previous work (Figure 1) [17]. In each experiment, changes in the immune cell infiltrate were compared with age‐matched PBS‐injected mice. We also observed an increase in neutrophils in the contralateral mammary gland 18 h after subcutaneous injection, although at reduced levels to that observed in the underlying mammary gland. No other significant differences were observed (Supplemental Figure S7).

**FIGURE 6:**
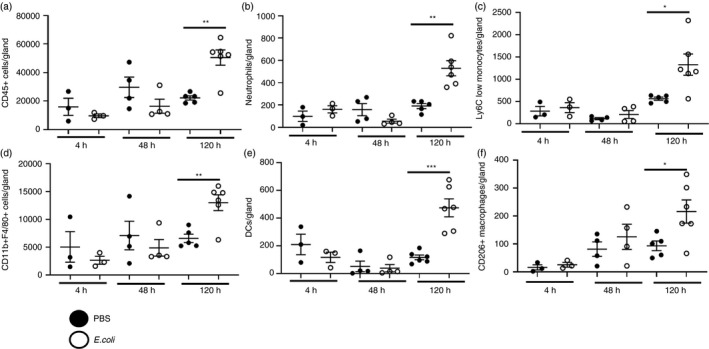
Myeloid cells are recruited to the mammary gland during intraperitoneal infection. Flow cytometry was used to determine the number of a) CD45+ cells, b) neutrophils, c) Ly6C low monocytes, d) CD11b+F4/80+ macrophages, e) Dendritic cells and f) CD206+ macrophages within the mammary gland after intraperitoneal challenge with either PBS (denoted by black circles) or 1 × 10^6^ CFU *E*. *coli* strain CFT073 (white circles), for 4 h (*n* = 3 per group), 2 days (*n* = 4 per group) and 5 days (PBS, *n* = 5, *E*. *coli*, *n* = 6). Significantly different results are indicated. Error bars represent SEM

To investigate whether myeloid cells are altered in the mammary gland during a live bacterial infection, 6‐ to 7‐week‐old mice were infected intraperitoneally with 1 × 10^6^ CFU of a uropathogenic strain of *E*. *coli* (CFT073). 5 days after infection of the peritoneum, the number of CD45+ cells within the mammary gland increased (Figure 6a). As observed during sterile inflammation, increased numbers of neutrophils, Ly6C low monocytes and CD11b+F4/80+ macrophages were observed (Figure 6b–d). We also detected increased numbers of DCs and CD206+ macrophages during infection, which was not seen during sterile challenge (Figure 6e,f).

### Mammary gland structures are altered during infection and inflammation

**FIGURE 7:**
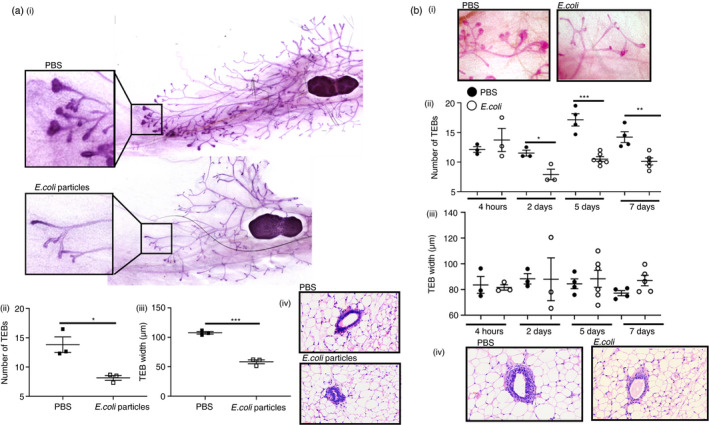
Mammary gland structures are altered during infection and inflammation. a) i) Representative carmine alum whole mount images (10×) of pubertal virgin mammary glands 3 days after intravenous (i.v) challenge with PBS or 200 µg *E*. *coli* particles (ECP). ii) The number of terminal end buds (TEB) was determined as the average number from at least 2 individual fields of view (FOV) (5×) per gland and iii) the average width of all TEBs was determined from at least 2 F.O.V (5×) per gland, 3 d after i.v challenge with PBS (denoted by black squares) or ECP (white squares) (*n* = 3 per group). iv) Representative 40× image of H&E‐stained mammary glands after i.v challenge with PBS or ECP. b) i) Representative carmine alum images (5×) of pubertal virgin mammary glands 2 days after intraperitoneal challenge with PBS or 1 × 10^6^ CFU *E*. *coli* CFT073. ii) The number of TEBs and iii) average width of all TEBs after i.p challenge with either PBS (black circles) or 1 × 10^6^ CFU *E*. *coli* strain CFT073 (white circles), after 4 h (*n* = 3 per group), 2 days (*n* = 3 per group), 5 days (PBS, *n* = 4, *E*. *coli*, *n* = 6) and 7 days (PBS, *n* = 4, *E*. *coli*, *n* = 5). iv) Representative 40× image of H&E‐stained mammary glands 7 days after i.p challenge with PBS or *E*. *coli*. Significantly different results are indicated. Error bars represent SEM

We next investigated whether inflammation of the mammary gland during puberty altered development of the gland. We analysed carmine alum stained whole mounts of mammary glands from pubertal WT mice challenged with either PBS, 200 µg ECP or 1 × 10^6^ CFU *E*. *coli* CFT073 (Figure 7). TEBs are highly proliferative structures within the pubertal mammary gland which give rise to the ductal epithelial network within the gland. Strikingly we found that 3 days after intravenous challenge with 200 µg of ECP, the average number and width of terminal end buds was markedly reduced (Figure 7a). We also investigated the effect of live bacterial infection of the peritoneum on TEB formation in the mammary gland and found that TEBs were reduced 2, 5 and 7 days after infection (Figure 7bii). However, the morphology of the TEB was not affected during peritoneal infection (Figure 7biii,iv). No differences were observed in the rate of mammary gland growth in terms of ductal elongation and branched area, following sterile inflammation or peritoneal bacterial infection (Supplemental Figure S8).

## DISCUSSION

Previously our understanding of immune system diversity within the mammary gland in normal development has been limited. Here, we provide a detailed analysis of the myeloid cell landscape in the mouse mammary gland at key time points in development and in murine milk. Macrophages are a particularly heterogeneous cell type within the mammary gland. Recent studies have revealed novel subsets such as CD206+ macrophages which promote branching in late puberty [17, 21], and ductal macrophages which cover the epithelium in lactation and involution to facilitate remodelling [18]. Recently, a population of Lyve‐1+ resident macrophages have also been identified which are important in maintaining ECM homeostasis within the mammary gland [22]. Here, we have used multi‐parameter flow cytometry to reveal a further three macrophage subsets within the mammary gland CD11b+CD11c+, CD11b+CD11c− and CD11b−CD11c−. Further studies to investigate whether these macrophages have overlapping, or distinct functions could reveal important insight into macrophage control of mammary gland development and surveillance.

In addition, we have investigated other important myeloid cell types in the mammary gland throughout development and in inflammation. We believe this study represents the first full investigation of myeloid cells throughout each of these key developmental stages. Although not exclusively, a proportion of mammary gland macrophages, at rest, and in inflammation, are derived from monocytes in the bone marrow [23]. Here, we reveal that both Ly6C high and Ly6C low monocytes are present in the developing mammary gland at each stage, increasing significantly between early and late puberty. In the absence of CCR2, both subtypes are depleted in the mammary gland. Monocytes are also identified in established murine milk and likely contribute to neonatal immune protection. We have also defined for the first time 2 types of eosinophil based on F4/80 positivity. Type 1 (F4/80+) eosinophils increase in late puberty and are recruited through a CCR2‐dependent mechanism. In contrast, type 2 (F4/80+) eosinophils are recruited through CCR3 and are unchanged in puberty. To our knowledge, our study is the first to describe the presence of small populations of neutrophils and DCs in the mammary gland of mice during virgin and pregnant development. Further studies are required to define their functions at these key developmental time points. As has been reported previously, neutrophils are found at higher numbers in early involution and are found at high levels in milk where they are thought to contribute to innate immune defence in the neonate [8, 12].

We also carried out a detailed analysis of myeloid cell recruitment to the gland in individual and compound iCCR‐deficient mice which lack all 4 receptors. We are able to confirm that CCR2 is the dominant monocyte receptor in the mammary gland, as has been observed throughout the body [15]. Notably, reduced recruitment of myeloid cells in iCCR−/− mice corresponded with the cell types reduced in individual receptor‐deficient mice. This suggests that there are no additional combinatorial effects of multiple receptor deficiency. Here, we have studied each individual receptor and the compound iCCR‐deficient mice at a defined stage of virgin development. As cell recruitment during mammary gland development is stage specific, comparisons across an extensive time course will be important to fully understand the role of chemokine receptors throughout mammary gland development.

Importantly, in this study we have revealed that local subcutaneous inflammation and bacterial infection at a distant site within the body can have profound effects on the immune cells present in the mammary gland. For the first time, we have been able to show that an inflammatory environment directly affects developmental structures within the mammary gland. TEBs drive the formation of the ductal epithelial network during puberty. In this study, during inflammation and infection, the number of TEBs decreases. It is important to note that in addition to immune cell recruitment, changes in inflammatory cytokines and levels of circulating hormones in response to inflammation and infection may contribute to the differences observed. Of note, a recent study has shown that either intraductal injection or colonization of the gut with enterotoxigenic *Bacteroides Fragilis* (ETBF) led to similar levels of bacteria within the mammary gland [24]. In ETBF gut colonized mice, the TEBs were enlarged, further indicating that infection can alter these structures within the mammary gland [24].

It has been shown that early breast development leads to higher risks of breast cancer in later life [25], and women with dense epithelial networks in the breast are more likely to develop breast cancer [26]. Thus, the potential to manipulate the immune system to delay branching in puberty could have significant health benefits. There is evidence to suggest that increased infections in early life lead to a delay in female puberty [27]. This previously unknown effect of inflammatory burden on mammary structures during puberty could have important implications for understanding how pubertal development is controlled.

## METHODS

### Animals

Animal experiments were carried out under a UK Home Office Project Licence and conformed to the animal care and welfare protocols approved by the University of Glasgow. C57BL/6 mice, CCR1−/−, CCR2−/−, CCR3−/−, CCR5−/− and iCCR−/− [28], and *MacGreen* mice [29] were bred at the specific pathogen‐free facility of the Beatson Institute for Cancer Research. All mice used for experiments in this study were female. Oestrous stage was determined by visual inspection as described by Byers et al [30]. Forced involution was achieved by removing pups from the mother at lactation day 7.

### Mammary gland digestion

The inguinal lymph node was removed, and the fourth inguinal mammary gland was chopped coarsely. Enzymatic digestion with 3 mg/ml collagenase type 1 (Sigma) and 1·5 mg/ml trypsin (Sigma) in 2 ml Leibovitz L‐15 medium (Sigma) was carried out in a 37°C shaking incubator at 200 rpm for 1 h. Tissue was shaken for 10 s before 5 ml of L‐15 medium supplemented with 10% foetal calf serum (Invitrogen) was added. Centrifugation was carried out at 400 g for 5 min. Red Blood Cell Lysing Buffer Hybri‐Max (Sigma) was applied for 1 min prior to washing in PBS containing 5 mM EDTA. Cells were then resuspended in 2 ml 0·25% Trypsin‐EDTA (Sigma) and incubated for 2 min at 37°C. Next, 5 ml of L‐15 containing 1 μg/ml DNase1 (Sigma) was added for 5 min at 37°C. L‐15 containing 10% FCS was then added to stop the reaction, and cells were filtered through a 40‐μm cell strainer. Finally, cells were washed in FACS buffer (PBS containing 1% FCS and 5 mM EDTA). Milk was obtained by removing mammary glands from lactating mice at day 7. Pups were removed from the mother at least 4 h prior to the dissection of lactating mammary glands to allow “leaked” milk to be collected. Glands were placed intact in FACS buffer, and milk was collected, washed and stained.

### Flow cytometry

Antibodies were obtained from BioLegend and used at a dilution of 1:200: CD45 BV605 (30‐F11), CD11b BV785 (M1/70), F4/80 PE‐Cy7 (BM8), SiglecF BV421 (S17007L), Ly6C APC‐Fire 750 (HK1·4), CD11c BV650 (N418), MHCII AF700 (M5/114·15·2), CD64 AF647 (X54‐5/7·1), MerTK FITC (2B10C42) and CD206 PerCP‐Cy5·5 (C068C2) for 30 min at 4°C. Ly6G BUV395 (1A8) and CD103 BUV805 (M290) were obtained from BD Bioscience. Dead cells were excluded using Fixable Viability Dye eFluor 506 (Thermo Fisher). Flow cytometry was performed using a Fortessa (BDBiosciences) and analysed using FlowJo V10.

### Sterile Inflammation of the mammary gland

Female mice between 6–7 weeks of age were injected subcutaneously with 500 µg, or intravenously with 200 µg of FITC labelled *E*. *coli* (K‐12 strain) Bioparticles in 200 µl PBS (Thermo). After a defined number of days, mice were culled and mammary glands were excised and processed for whole mount and cellular analysis.

### Peritoneal bacterial infection

Female mice between 6–7 weeks of age were injected intraperitoneally with 1 × 10^6^ CFU of *E*. *coli* (CFT073 strain). Bacteria were grown overnight in Luria‐Bertani medium, before being sub‐cultured and grown to log phase for injection (OD_600_ = 0·5, 5 × 10^8^ CFU/ml). Mice were monitored for weight loss and clinical signs of infection. Mice were culled, and mammary glands were excised and processed for whole mount and cellular analysis.

### Carmine alum whole mount

Carmine alum whole mounts were carried out as previously described [16, 17]. Briefly, fourth inguinal mammary glands were fixed in 10% neutral‐buffered formalin (NBF) (Leica) overnight at 4°C. Glands were dehydrated for 1 h in distilled water, then 1 h 70% ethanol and 1 h 100% ethanol before incubation in xylene overnight (VWR international). Rehydration was achieved by 1‐h incubation in 100% ethanol, 70% ethanol and distilled water, before staining with Carmine Alum solution at room temperature overnight (0·2% (w/v) carmine and 10 mM aluminium potassium sulphate (Sigma)). Tissue was dehydrated again and incubated overnight in xylene. Glands were then mounted with DPX (Leica), and 10× magnification stitched brightfield images were obtained using an EVOS FL auto2 microscope (Thermo Fisher). 5× brightfield images were obtained using the Zeiss Axio Imager M2 with Zen 2012 software. TEBs were counted as the average from at least 2 F.O.V. from each whole mount. All samples were blinded before measurements were taken.

### Statistical analysis

Data were analysed using GraphPad Prism 8.1.2. Normality was assessed using Shapiro–Wilk and Kolmogorov–Smirnov tests. For normally distributed data, two‐tailed, unpaired t‐tests were used. Where data were not normally distributed, Mann–Whitney tests were used. Multiple comparison analysis was carried out using an ANOVA with Tukey's post‐test (normal distribution) or Kruskal–Wallis (not normal distribution). Significance was defined as *p* < 0·05 *. Error bars indicate standard error of the mean (SEM).

## CONFLICTS OF INTEREST

The authors declare no competing interests.

## AUTHOR CONTRIBUTIONS

GJW conceived the study, performed experiments, analysed data and wrote the paper. AF and FV performed experiments. GJG conceived the study and wrote the paper.

## Supporting information

Supplementary Material
