# Supplementary material for: Diverse myeloid cells are recruited to the developing and inflamed mammary gland
Source: Immunology. 2021 Nov 30;165(2):206–18. doi: 10.1111/imm.13430 (PMC10357480; doi:10.1111/imm.13430)
Supplement: Supplementary file 1 — Supplementary Material [file IMM-165-206-s001.pdf]

Supplemental Information

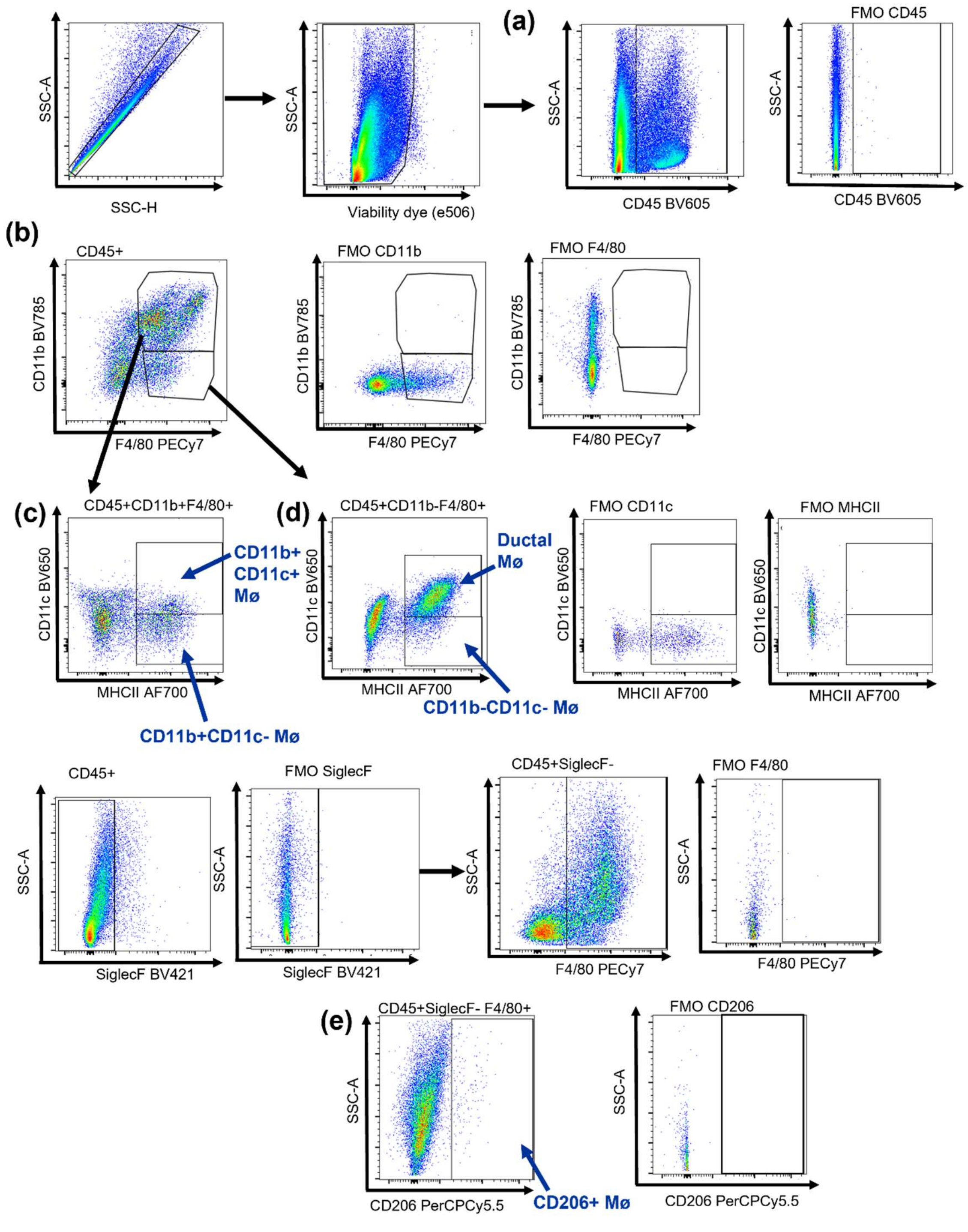

Supplemental Figure 1: Gating strategy to define macrophage subsets in the mammary gland. Flow cytometry was carried out to identify cell types in the mammary gland. Single cells were gated, dead cells were excluded, and **a)** CD45+ immune cells were identified. Populations were then expressed as a percentage of CD45+ cells, including; **b)** CD11b+F4/80+ macrophages, **c)** CD11b+F4/80+CD11c+ and CD11b+F4/80+CD11c+ macrophages, **d)** CD11b-F4/80+CD11c+ ductal macrophages and CD11b-F4/80+CD11c- macrophages, and **e)** SiglecF-F4/80+CD206+ macrophages. 'Fluorescence minus one' (FMO) plots represent gating controls.

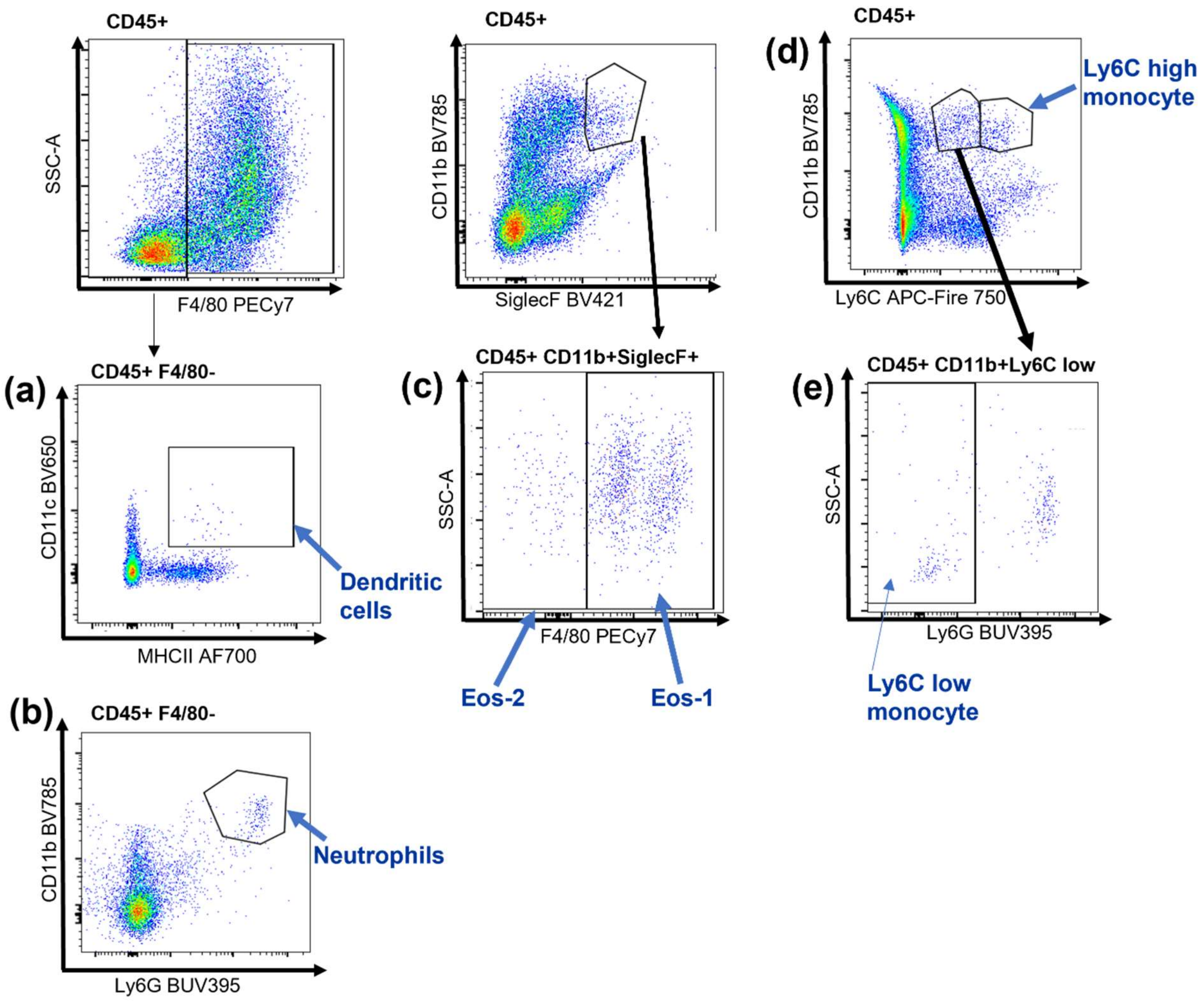

Supplemental Figure 2: Gating strategy to define myeloid cells in the mammary gland. Flow cytometry was carried out to identify cell types in the mammary gland. Single cells were gated, dead cells were excluded, and CD45+ immune cells were identified. Populations were then expressed as a percentage of CD45+ cells, including; **a)** F4/80-CD11c+MHCII+ dendritic cells, **b)** F4/80-CD11b+Ly6G+ neutrophils, **c)** type 1 (F4/80+) and type 2 (F4/80-) eosinophils, **d)** CD11b+Ly6C high monocytes and **e)** CD11b+Ly6C low Ly6G- monocytes.

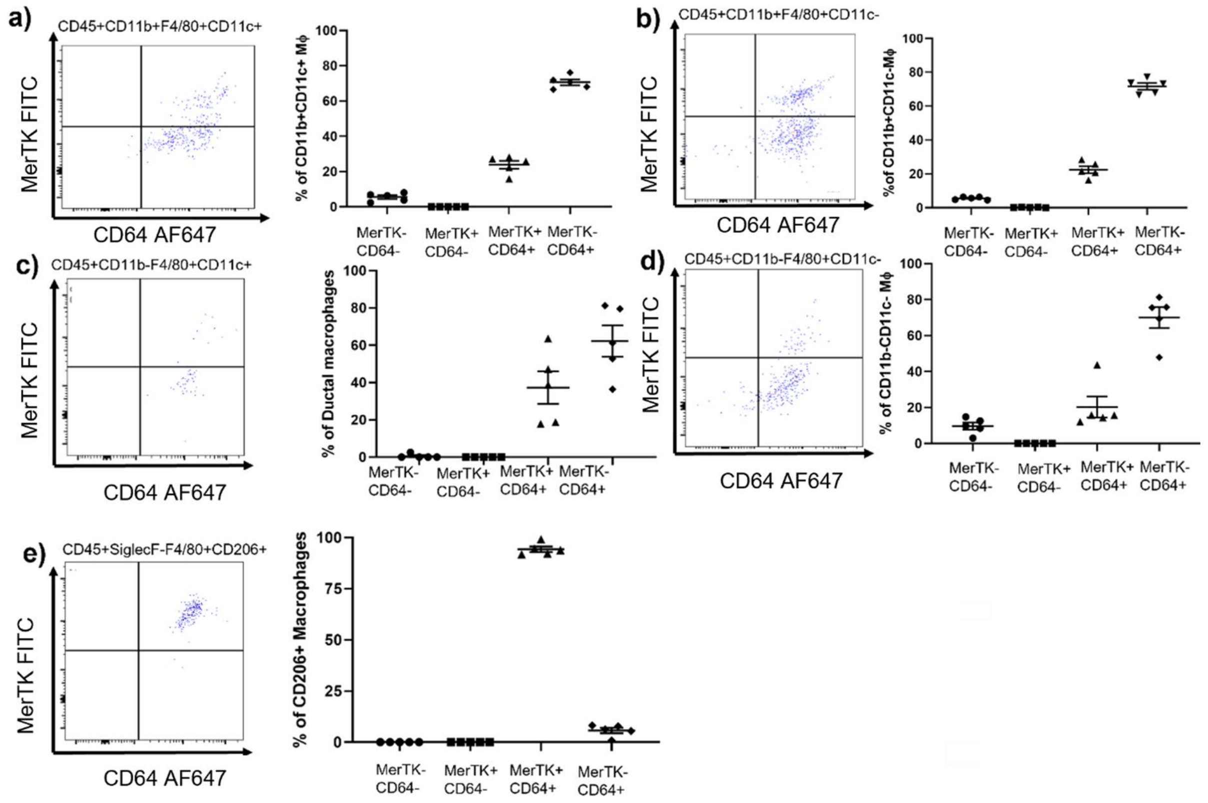

Supplemental Figure 3: CD64 and MerTK expression by mammary gland macrophages. Flow cytometry of **a)** CD11b+CD11c+, **b)** CD11b+CD11c-, **c)** Ductal CD11b- CD11c+, **d)** CD11b-CD11c- and **e)** CD206+ mammary gland macrophages to reveal the expression of CD64+ and MerTK+ by each macrophage subtype in 7 weeks old virgin mice (n=5). Error bars represent S.E.M.

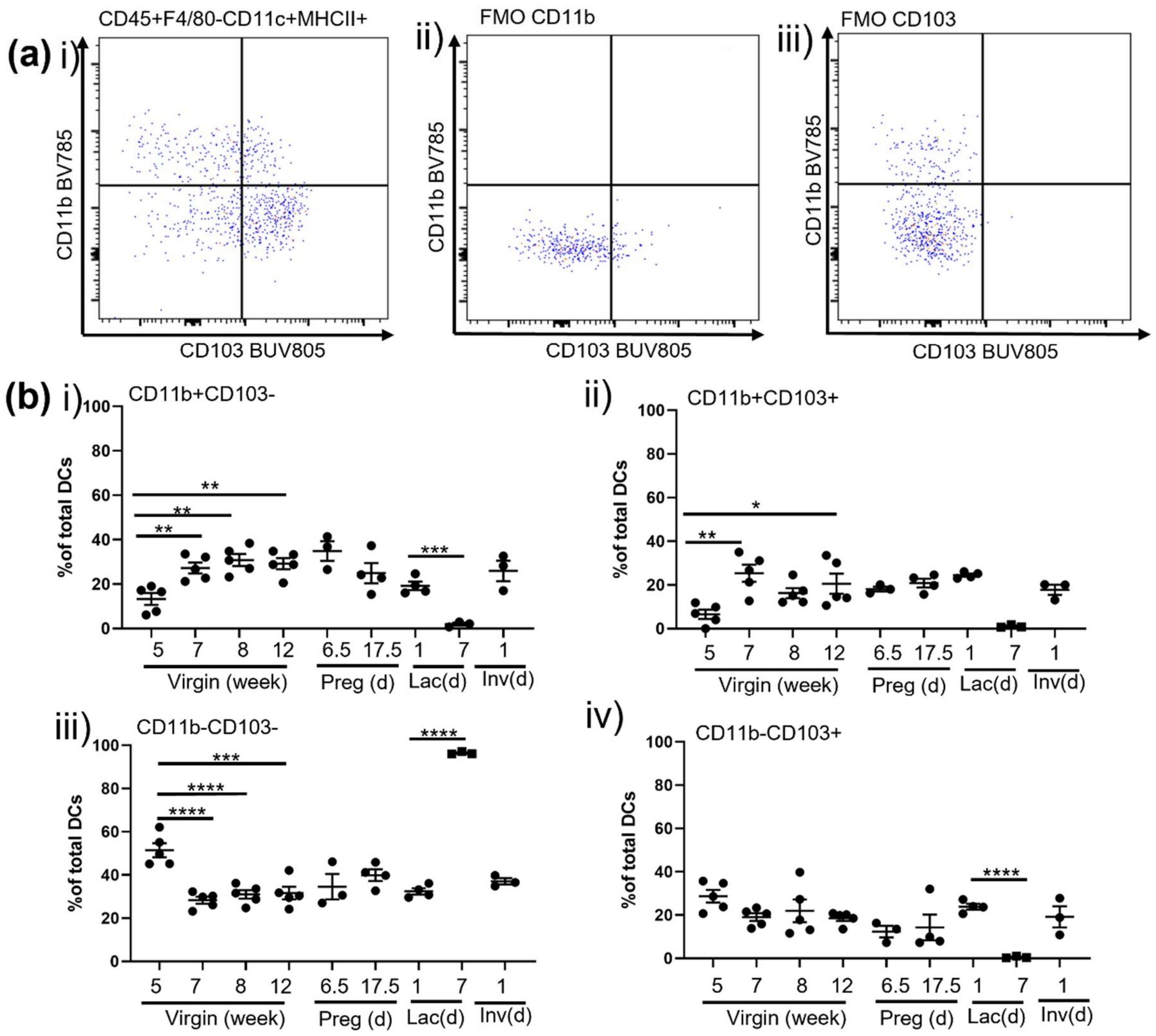

Supplemental Figure 4: Dendritic cell heterogeneity within the mammary gland. **a) i)** Flow cytometry revealed CD11b and CD103 expression within the total dendritic cell population (F4/80- CD11c+ MHCII+) of 8 week old virgin mice. 'Fluorescence minus one' controls for **ii)** CD11b and **iii)** CD103. **b)** The percentage of the total DC population which are **i)** CD11b+CD103- **ii)** CD11b+CD103+, **iii)** CD11b-CD103- and **iv)** CD11b-CD103+ at key time points in virgin development at 5, 7, 8 and 12 weeks (n=5 per group), pregnancy day 6.5 (n=3) and 17.5 (n=4), lactation day 1 (n=4) and 7 (n=3) and involution day 5 (n=3). Statistically significant results are indicated. Error bars represent S.E.M.

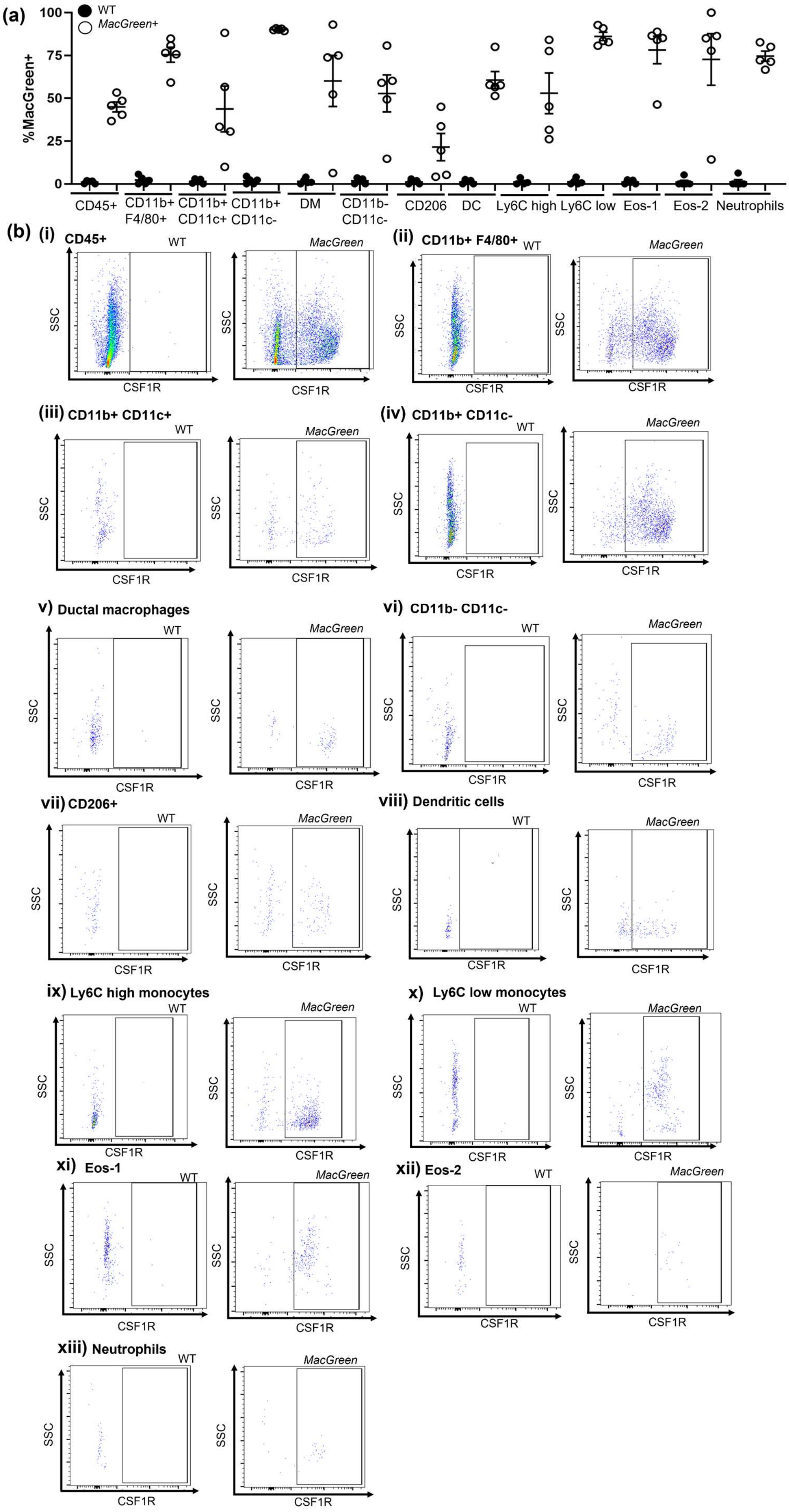

(a)
WT
MacGreen+
%MacGreen+
100
75
50
25
0
CD45+
CD11b+ F4/80+
CD11b+ CD11c+
CD11b+ CD11c-
DM
CD11b- CD11c-
CD206
DC
Ly6C high
Ly6C low
Eos-1
Eos-2
Neutrophils
(b) (i) CD45+
(ii) CD11b+ F4/80+
(iii) CD11b+ CD11c+
(iv) CD11b+ CD11c-
v) Ductal macrophages
vi) CD11b- CD11c-
vii) CD206+
viii) Dendritic cells
ix) Ly6C high monocytes
x) Ly6C low monocytes
xi) Eos-1
xii) Eos-2
xiii) Neutrophils
WT
MacGreen
SSC
CSF1R

Supplemental Figure 5: CSF1R expression in the mammary gland. a) Flow cytometry of WT and *MacGreen* (CSF1R GFP reporter) transgenic mice to reveal the percentage of CSF1R+ cells within each cell type, CD45+ cells, CD11b+F4/80+ macrophages, CD11b+CD11c+F4/80+ MHCII+ macrophages, CD11b+CD11c- F4/80+ MHCII+ macrophages, Ductal macrophages (DM), CD11b-CD11c-F4/80+MHCII+ macrophages, CD206+ macrophages, total dendritic cells (DC), Ly6C high monocytes, Ly6C low monocytes, eosinophils type 1, eosinophils type 2, and neutrophils (n=5 per group). **b) i)** Representative flow cytometry plots from each cell type within WT and *MacGreen* mammary glands, **i)** CD45+ cells, **ii)** CD11b+F4/80+ macrophages, **iii)** CD11b+CD11c+F4/80+MHCII+ macrophages, **iv)** CD11b+CD11c-F4/80+MHCII+ macrophages, **v)** Ductal macrophages, **vi)** CD11b-CD11c-F4/80+MHCII+ , **vii)** CD206+ macrophages, **viii)** Dendritic cells, **ix)** Ly6C high monocytes, **x)** Ly6C low monocytes, **xi)** eosinophils type 1, **xii)** eosinophils type 2, and **xiii)** neutrophils**.** Error bars represent S.E.M.

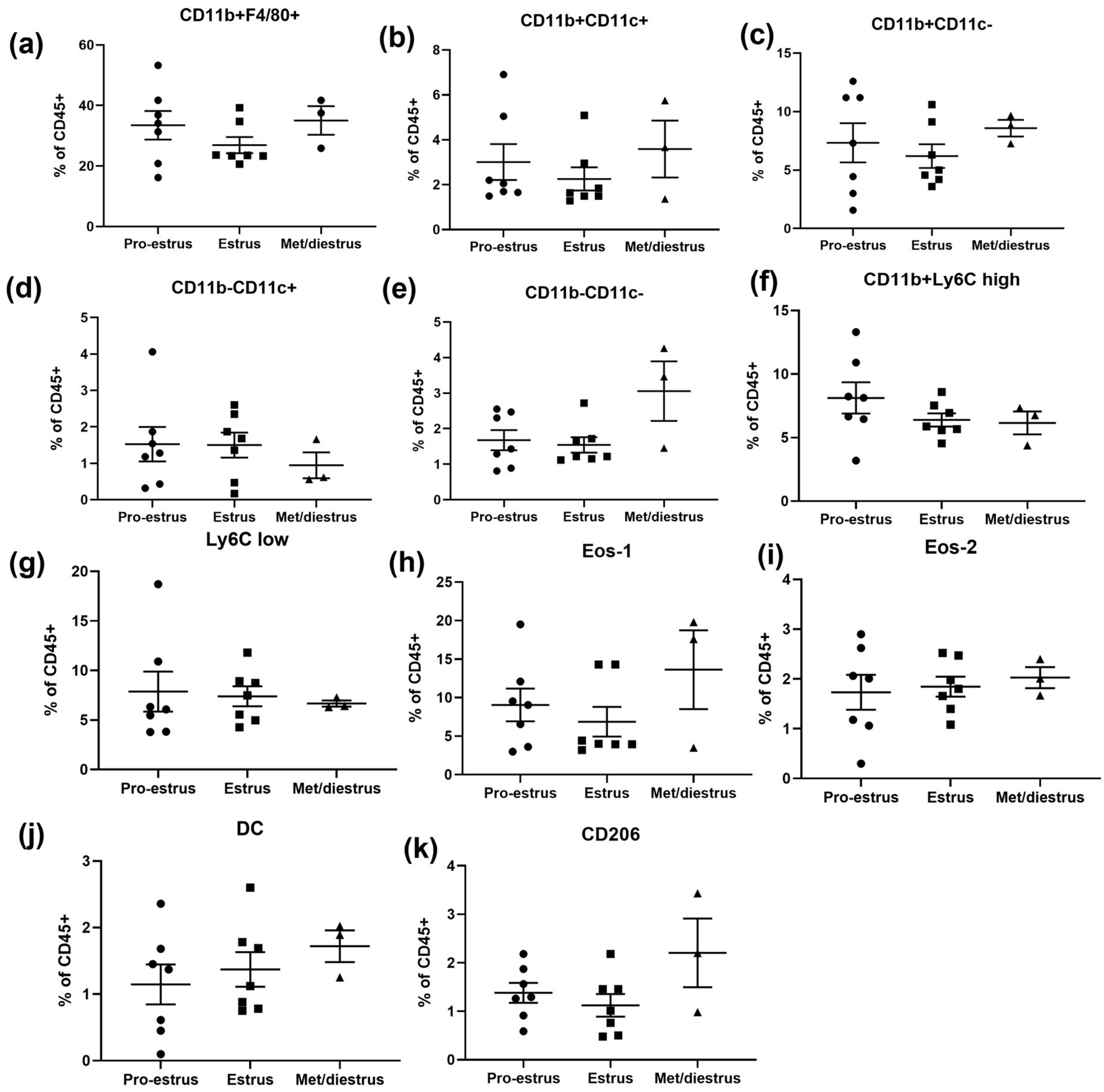

Supplemental Figure 6: Estrous stage does not alter the frequency of cell types. Flow cytometry of **a)** CD11b+F4/80+, **b)** CD11b+CD11c+, **c)** CD11b+CD11c, **d)** Ductal macrophages, **e)** CD11b-CD11c-, **f)** CD11b+ Ly6C high monocytes, **g)** Ly6C low monocytes, **h)** eosinophils type 1, **i)** eosinophils type 2 and **k)** CD206+ mammary gland macrophages does not reveal any changes in frequency of CD45+ cells within the mammary glands of 7 week old virgin mice during pro-estrous (n=7), estrous (n=7), and metestrous/ diestrous (n=3). Error bars represent S.E.M.

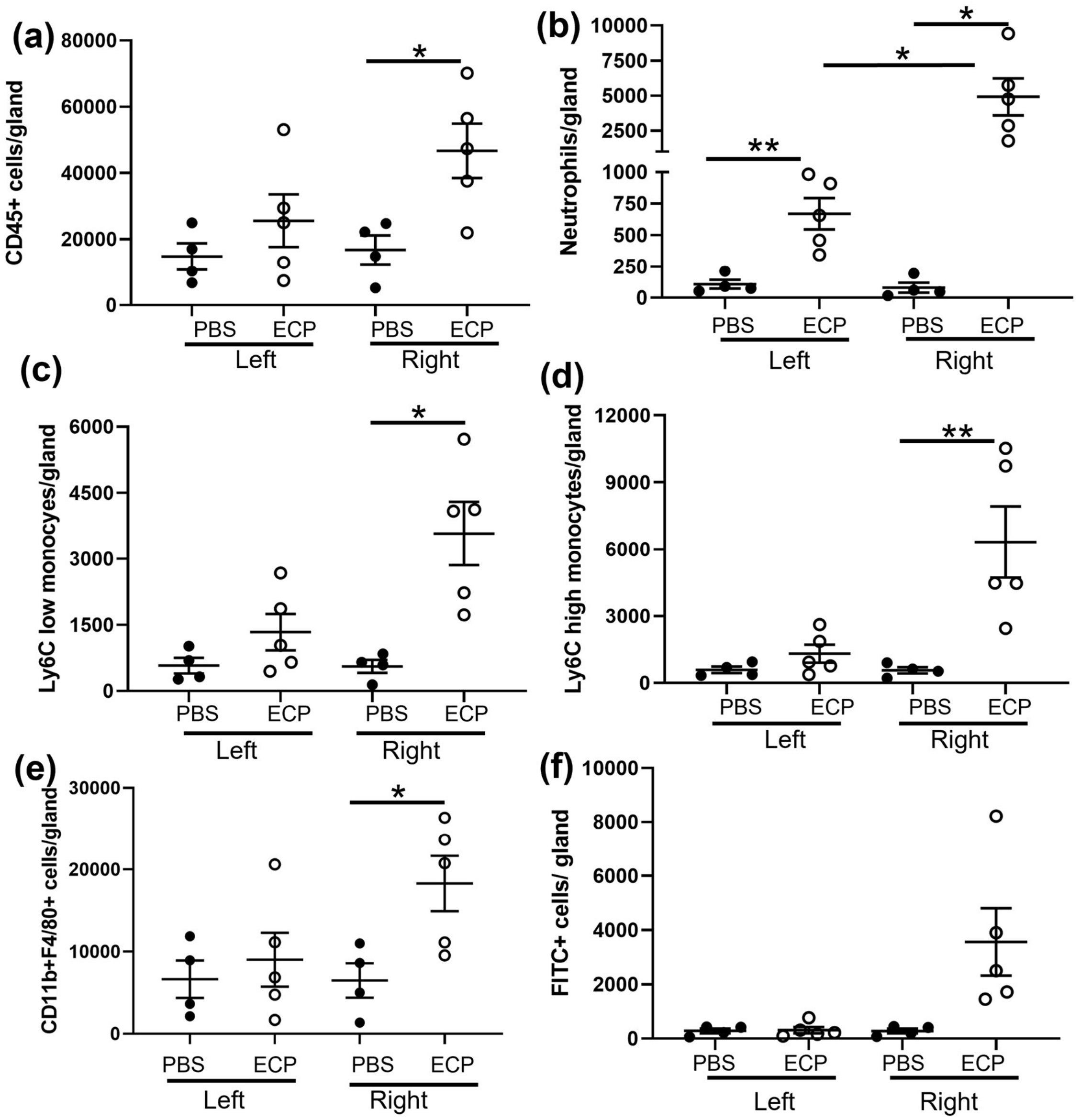

Supplemental Figure 7: Myeloid cell recruitment to the underlying and contralateral mammary gland after subcutaneous challenge. Flow cytometry was used to determine the number of **a)** CD45+ cells **b)** neutrophils, **c)** Ly6C low monocytes, **d)** Ly6C high monocytes, **e)** CD11b+F4/80+ macrophages and **f)** cells bound by FITC labelled *E.coli* particles (ECP) within the right (underlying) and left (contralateral) mammary gland 18h after subcutaneous challenge with either PBS (denoted by black circles) or 500 µg of ECP (white circles), (PBS, n=4, ECP, n=5). Significantly different results are indicated. Error bars represent S.E.M.

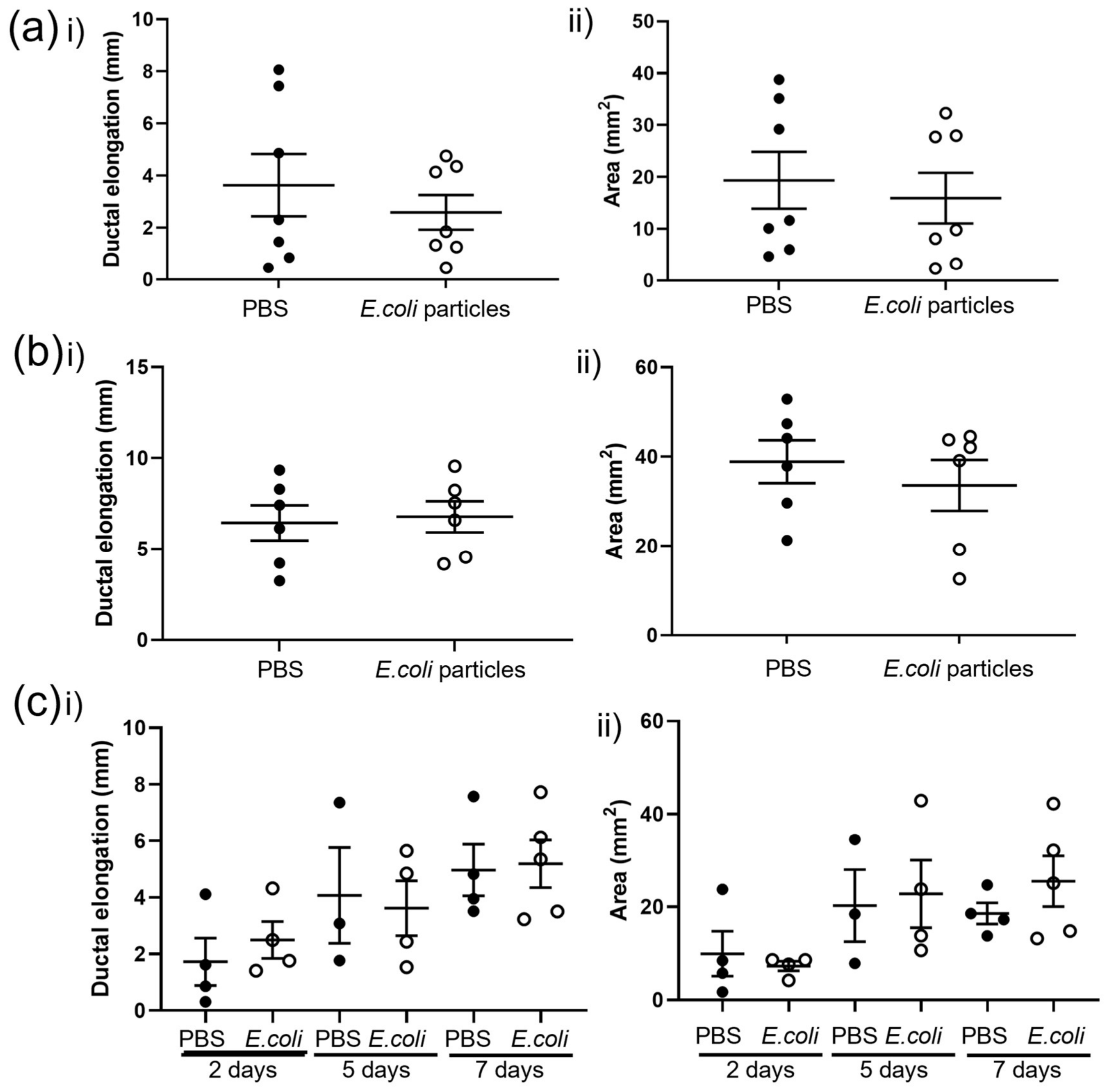

Supplemental Figure 8: Ductal elongation and branched area is unchanged after inflammation and infection. Measurements were taken **a)** 3 days after intravenous injection of either PBS or 200 µg *E.coli* particles (ECP) (n=7 per group), **b)** 5 days after subcutaneous injection of either PBS or 500 µg ECP (n=6 per group), **c)** 2, 5 and 7 days after intraperitoneal infection with *E.coli* CFT073 (2 days, PBS, *E.coli* n=4, 5 days, PBS n=3, *E.coli* n=4, 7 days, PBS, n=4, *E.coli* n=5). **i)** Ductal elongation was measured from the middle of the inguinal lymph node to the furthest edge of ductal outgrowth, and **ii)** the area of branching from the inguinal lymph node was measured using Image J. Error bars represent S.E.M.

Supplemental Table 1: Statistical analysis comparing immune cells throughout development.

Cell type	CD11b+F4/80+	CD206+	Ductal macrophage	CD11b+ CD11c+	CD11b+ CD11c-	CD11c- CD11b-	Ly6C high monocytes	Ly6C low monocytes	Eos-1	Eos-2	Neutrophils	Total DC
**Virgin**												
5 weeks vs. 6.5 weeks	**	**	ns	*	ns	ns	**	**	**	ns	ns	ns
5 weeks vs. 7 weeks	***	**	ns	ns	ns	ns	ns	ns	ns	ns	ns	ns
5 weeks vs. 8 weeks	*	ns	ns	ns	*	*	ns	ns	ns	ns	ns	ns
5 weeks vs. 12 weeks	ns	ns	ns	ns	ns	****	ns	ns	ns	ns	ns	ns
5 weeks vs. 28 weeks	ns	ns	ns	***	ns	ns	ns	ns	ns	**	ns	ns
6.5 weeks vs. 7 weeks	ns	ns	ns	ns	ns	ns	*	ns	ns	ns	ns	ns
6.5 weeks vs. 8 weeks	ns	ns	ns	*	ns	***	**	*	ns	ns	ns	*
6.5 weeks vs. 12 weeks	ns	*	ns	ns	ns	****	***	ns	ns	ns	ns	ns
6.5 weeks vs. 28 weeks	ns	ns	ns	ns	ns	ns	***	***	*	*	ns	**
7 weeks vs. 8 weeks	ns	ns	*	ns	ns	**	ns	ns	ns	ns	ns	ns
7 weeks vs. 12 weeks	ns	*	ns	ns	ns	****	ns	ns	ns	ns	*	ns
7 weeks vs. 28 weeks	ns	ns	ns	ns	ns	ns	ns	ns	ns	***	ns	ns
8 weeks vs. 12 weeks	ns	ns	ns	ns	ns	**	ns	ns	ns	ns	ns	ns
8 weeks vs. 28 weeks	ns	ns	**	***	ns	ns	ns	ns	ns	ns	ns	ns
12 weeks vs. 28 weeks	ns	ns	ns	**	ns	****	ns	ns	ns	ns	ns	ns
**Pregnant**												
P6.5 vs. P13.5	ns	ns	ns	ns	ns	ns	ns	ns	ns	ns	ns	ns
P6.5 vs. P15.5	ns	ns	ns	ns	ns	ns	ns	ns	ns	ns	ns	ns
P6.5 vs. P17.5	ns	ns	ns	ns	ns	ns	ns	ns	ns	ns	ns	ns
P13.5 vs. P15.5	ns	ns	ns	ns	ns	ns	ns	ns	ns	ns	ns	ns
P13.5 vs. P17.5	ns	ns	ns	ns	ns	ns	ns	*	ns	ns	ns	ns
P15.5 vs. P17.5	ns	ns	ns	ns	ns	ns	ns	ns	ns	ns	ns	ns
**Lactation**												
Lac d1 vs d7	**	ns	***	**	**	ns	**	**	ns	*	ns	*
**Involution**												
Inv d1 vs d5	***	*	***	*	**	ns	*	***	*	ns	*	**
